# Dielectric Energy Storage Performance of Reductive Polyaniline/Polyethylenimine All-Organic Composite Films with Tunable Molecular Weight and Chain Structure

**DOI:** 10.3390/polym18091080

**Published:** 2026-04-29

**Authors:** Yuanfeng Li, Jingyu Lin, Ruihang Zhang, Xinyan Zhang, Shumu Zhou, Qixin Zhuang, Peiyuan Zuo

**Affiliations:** Key Laboratory of Advanced Polymeric Materials of Shanghai, School of Materials Science and Engineering, East China University of Science and Technology, Shanghai 200237, China

**Keywords:** polymer nanocomposite dielectrics, reduced polyaniline (R-PANI), polyetherimide, discharge energy density, wide-temperature energy storage

## Abstract

High-efficiency energy storage technologies have become particularly crucial with the ever-increasing demand for energy in recent years. Research on polymer nanocomposite dielectric materials has emerged as a prominent focus. Particularly, there is an urgent demand for the development of advanced dielectric film materials that exhibit superior energy storage performance over a wide temperature range. To this end, this study aims to investigate the effect of the molecular weight of reduced polyaniline (R-PANI) on the dielectric properties of all-organic composite films based on high-temperature-resistant polyetherimide (PEI). All-organic R-PANI/PEI composite films were fabricated by blending PEI matrix with R-PANI of varying molecular weights. Through combined density functional theory (DFT) calculations and experimental measurements, the blocking mechanism of R-PANI on charge carrier migration within the composite films was elucidated, showing a significant enhancement in the discharge energy density of PEI polymers while maintaining high charge–discharge efficiency. With charge–discharge efficiency maintained above 95%, R-PANI3/PEI achieved a discharge energy density of 2.36 J cm^−3^ at room temperature, nearly double that of pristine PEI (1.2 J cm^−3^). At 150 °C, the 1.0 wt% R-PANI3/PEI composite film retained a discharge energy density of 2.27 J cm^−3^ with a charge–discharge efficiency of 89.2%, outperforming pure PEI (1.1 J cm^−3^, 85.1%). These findings provide a new strategy for the design of all-organic composite dielectric films and demonstrate the potential of R-PANI in the application of high-performance capacitors and electrical energy storage.

## 1. Introduction

As the demand for energy continues to rise across various fields [1,2], the development and utilization of renewable energy have become strategically important [3]. However, the intermittent and unstable nature of these renewable sources highlights the critical need for efficient energy storage technologies. To date, a variety of electrical energy storage devices have been developed, including electrochemical capacitors, solid oxide fuel cells (SOFCs), batteries, superconducting magnetic energy storage (SMES), and dielectric capacitors [4,5]. Each of these energy storage solutions presents distinct trade-offs in terms of energy density, power density, and charge–discharge efficiency [6,7,8,9,10]. Unlike batteries, fuel cells, and supercapacitors, the charge–discharge processes in dielectric capacitors are purely physical, allowing for safe and ultrafast energy storage and release on the microsecond timescale. This makes dielectric capacitors indispensable for applications in pulsed power supplies and voltage stabilization systems.

Dielectric capacitors, also known as electrostatic capacitors, are essential components in high-voltage, fast-charging technologies [11,12,13]. In recent years, the applications and market demand for polymer-based electrostatic capacitors have grown rapidly, and research on the use of nanotechnology to fabricate and enhance polymer nanocomposite dielectric materials has emerged as a prominent focus [14]. However, these capacitors typically exhibit limited energy density [15,16,17] and are often required to operate continuously under high-temperature conditions. Therefore, the development of advanced dielectric materials that can provide superior energy storage performance across a wide temperature range has become a critical priority [18,19,20,21].

To meet the demands for flexible, lightweight, high-energy storage and high temperature resistance in these applications, organic–inorganic composite materials have been extensively developed [22,23]. The structure–property relationships between the microstructures and dielectric performance of nanocomposite films fabricated via organic–inorganic blending have been explored. In these composite systems, a nanoscale interfacial region exists between the polymer matrix and the inorganic fillers. Many approaches have aimed to rationally design and manipulate this interfacial region, such as by using fillers with high specific surface area at ultra-low loadings to expand the interfacial polarization zone or introducing an organic dual-interfacial layer to enhance interfacial polarization and create a gradient in dielectric constant. Through methods such as these, it is possible to regulate the internal electric field distribution and improve the breakdown strength of the composite films. Consequently, the resulting composites exhibit superior dielectric properties that cannot be achieved with neat polymers. Nevertheless, organic–inorganic composite materials face challenges related to nanoparticle dispersion, which can compromise the flexibility of a polymer matrix. Although the introduction of organic interlayers improves interfacial compatibility, it also complicates the fabrication process. As a result, researchers have increasingly turned to all-organic polymer blending strategies [24,25], which not only simplify the preparation process but also enhance the materials’ potential for practical application and industrial scalability. For example, Zhang et al. [26] synthesized aromatic polythiourea (ArPTU) using a one-step method and blended it with polyetherimide (PEI) to create a series of all-organic composite films. Due to the strong polarity of ArPTU and its excellent compatibility with PEI, the resulting composite films exhibited improved energy storage density and charge–discharge efficiency compared to pure PEI.

**Figure 1:**
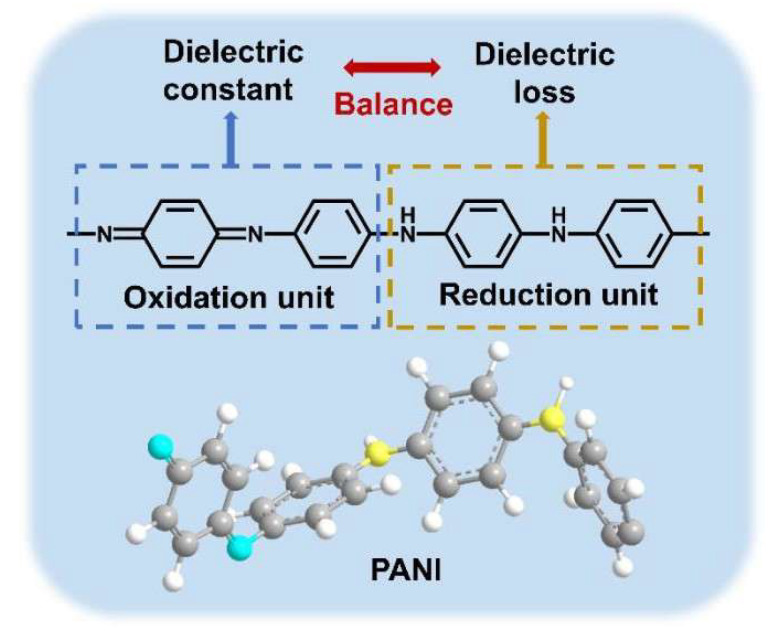
The structural formula of intrinsic polyaniline and its dielectric function diagram.

Polyaniline (PANI) is a well-known semiconductor polymer [27,28,29,30,31,32,33], which is usually synthesized from aniline via chemical oxidative polymerization [32,34,35,36]. MacDiarmid et al. [37] proposed that the intrinsic PANI model contains two structural units, i.e., oxidized and reduced units (Figure 1). The oxidized unit features a quinoid ring structure with strong polarizability, which readily generates a high polarization response under an electric field and thus increases the dielectric constant. The reduced unit exhibits weaker polarizability than the oxidized unit but hardly forms leakage pathways, leading to low dielectric loss. The oxidation state of PANI is determined by the ratio of quinoid to benzenoid ring structures in its backbone, and different oxidation states can be interconverted via redox reactions [38].

This study designed and prepared different reduced polyanilines (R-PANI) for all-organic blending with PEI, aiming to utilize the different responses of their oxidation and reduction units to modulate the dielectric properties of the PEI polymer. Density functional theory (DFT) calculations reveal that the benzene ring structures of PANI carry negative charges, which can generate electrostatic interactions with the positively charged benzene ring structures in the PEI main chain. Moreover, with the increase in benzene ring structures in the PANI main chain, it can generate stronger electronic barriers and deeper hole traps inside PEI to suppress carrier migration. Therefore, R-PANI, due to its relatively higher internal benzene ring content, can enhance the polarization of the PEI matrix while reducing dielectric loss. Through investigating the effect of R-PANI with different polymerization times on the dielectric properties of PEI, it was found that high-molecular-weight R-PANI can bring chain flexibility, enabling an easier polarization response and thus a larger interfacial region, so it has an advantage in improving the dielectric constant. However, excessively high filler content can lead to molecular chain entanglement and increased loss. In contrast, low-molecular-weight R-PANI, due to its easier dispersibility, can usually better exert an effect as an electronic barrier, reduce electric field concentration and defects, and thus play a greater role in reducing dielectric loss. At room temperature and with an electric field of 300 kV mm^−1^, the charge–discharge efficiency of 1 wt% R-PANI-3/PEI reaches 96%, with a discharge energy density of 2.36 J cm^−3^, significantly higher than the 1.2 J cm^−3^ of pure PEI. Furthermore, the all-organic thin film exhibits excellent dielectric and thermal stability. At 150 °C, the charge–discharge efficiency of pure PEI drops to 85.1%, with a discharge energy density of 1.11 J cm^−3^, while the discharge energy density of 1.0 wt% R-PANI-3/PEI reaches a high of 2.27 J cm^−3^, exhibiting an even higher charge–discharge efficiency (89.2%). The key novelty of this work lies in establishing a molecular weight engineering strategy within an all-organic system, moving beyond the conventional paradigm of simply blending PANI with inorganic fillers. Outputs in recent studies [27,28,29,30,31,32,33] mainly focus on non-all-organic composite systems (e.g., PANI/CuO, PANI/BaTiO_3_/rGO) to enhance dielectric constants; they often suffer from compromised breakdown strength, high dielectric loss, or severe high-temperature performance decay. Collectively, this work provides a new direction for the design of all-organic composite films and is expected to provide a reference for the design and development of composite film materials in this field.

## 2. Experimental

### 2.1. Materials and Reagents

All chemicals and Materials are presented as follows (Table 1).

### 2.2. Synthesis of PANI and R-PANI

In this section, polyaniline (PANI) was synthesized via chemical oxidation, and reduced polyaniline (R-PANI) was subsequently prepared through alkaline de-doping. First, 83.5 mL of concentrated hydrochloric acid was diluted to 1 mol/L in a 1 L volumetric flask, hereafter referred to as 1 M HCl. Aniline (2.1 g) was added to 240 mL of 1 M HCl in a round-bottom flask and maintained in an ice-water bath with mechanical stirring for 30 min to ensure thorough mixing, designated as solution A. Separately, 60 mL of 1 M HCl and 1.287 g of ammonium persulfate (APS) were magnetically stirred in a beaker for 10 min until fully dissolved, designated as solution B. Solution B was then added to solution A, and the mixture was stirred in an ice-water bath for 3, 6, 9, or 12 h. The solution was observed to gradually turn dark green. The resulting mixture was filtered through a sand core funnel and washed several times with deionized water until the filtrate reached a neutral pH. The solid products were dried in a vacuum oven at 60 °C for 12 h to remove residual moisture, yielding protonated PANI synthesized at different reaction times (designated as PANI-3, PANI-6, PANI-9, and PANI-12) with an approximate yield of 72%.

The synthesis procedure was repeated, and the wet PANI obtained from suction filtration was used directly for the preparation of R-PANI. Specifically, the PANI products were transferred to a three-necked flask with 200 mL of deionized water and dispersed uniformly under magnetic stirring. Subsequently, 10 mL of excess hydrazine hydrate (N_2_H_4_·H_2_O) was added to the system, which was then heated to 95 °C and maintained under condenser reflux for 6 h. The color of the mixture gradually changed from dark green to dark blue-purple. After cooling, the mixture was filtered using a sand core funnel and washed with water until the filtrate reached a neutral pH. Finally, the solid products were placed in a vacuum oven at 60 °C for 12 h to thoroughly remove residual moisture, yielding the dedoped R-PANI samples. These samples were designated as R-PANI-3, R-PANI-6, R-PANI-9, and R-PANI-12.

### 2.3. Fabrication of R-PANI/PEI All-Organic Composite Films

**Figure 2:**
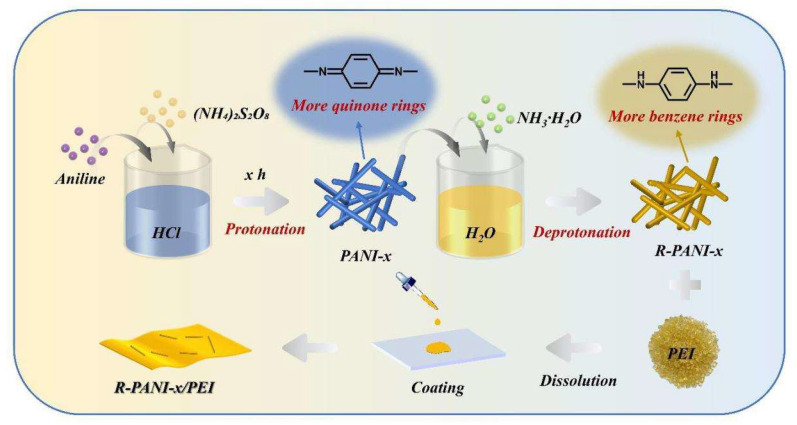
The preparation flowchart of R-PANI/PEI all-organic composite films.

First, 50 mg of PEI and R-PANI with mass fractions of 0, 0.25, 0.5, 0.75, and 1.0 wt% were weighed and dissolved in 1.2 mL of DMAc using magnetic stirring. The mixture was subsequently subjected to ultrasonication for 30 min prior to solution casting. Glass slides were pre-cleaned, rinsed with deionized water, and dried in a convection oven. The cleaned slides were then placed in a vacuum oven. The polymer blend solutions were then cast onto the glass slides in a vacuum oven and dried at 70 °C for 24 h under vacuum. The composite films were peeled from the glass slides in deionized water, immediately blotted dry, and dried again in a vacuum oven at 70 °C for 12 h to remove moisture. Finally, R-PANI/PEI all-organic composite films with the desired mass fractions were obtained (Figure 2).

### 2.4. Instrumentation and Characterization

The XRD patterns of PANI before and after reduction were recorded using a D/MAX 2550 VB/PC rotating anode X-ray diffractometer. The instrument was equipped with an 18 kW copper target (450 mA), and the scanning range was set from 10° to 80°. The FTIR spectra of PANI synthesized at different polymerization times were obtained using a Nicolet 6700 Fourier transform infrared spectrometer to obtain FTIR spectra. The morphology of PANI prepared with various polymerization times was observed and captured using a JEOL JEM-2100 high-resolution transmission electron microscope. The morphology of PANI and the cross-sectional structures of R-PANI/PEI composite films were investigated using a Hitachi S-4800 field emission scanning electron microscope. The molecular weights of reduced PANI samples obtained at different polymerization times were determined using a multi-detection gel permeation analyzer (DAWN HELEOS, Wyatt Technology, USA). DMF was used as the mobile phase with a flow rate of 1 mL min^−1^ at 25 °C, and the sample concentration was maintained at 3 mg mL^−1^. The composite films were cut into 1 cm diameter disks. Gold electrodes (3 mm diameter) were deposited on both sides of each sample using a Cressington 108 high-performance ion sputter coater (UK). The frequency-dependent (10^2^–10^6^ Hz) dielectric constant and dielectric loss, as well as their temperature-dependent (25–150 °C) variations at 1 kHz, were measured using a Concept 40 broadband dielectric spectrometer (Novocontrol Technologies GmbH & Co. KG, Germany). Unlike dielectric testing, breakdown strength measurements did not require gold electrode deposition. The breakdown strength was calculated based on the recorded film thickness and the applied voltage at the moment of breakdown, as measured by a CS2674AX high-voltage withstand tester. Following the same electrode preparation procedure as for dielectric testing, the displacement–electric field (D–E) hysteresis loops were measured at 10 Hz using a Radiant Precision Premier II ferroelectric analyzer on samples with sputtered gold electrodes. The energy storage density was subsequently calculated from the hysteresis loops. Leakage current density was measured for 1 cm diameter samples with 5 mm sputtered gold electrodes using a Radiant Technology measurement system under an electric field range of 0–200 kV mm^−1^, a frequency of 10 Hz, and a temperature of 150 °C. DFT calculations were performed using Gaussian 09W and visualized via GaussView 5.0. Structural optimizations for both PEI and PANI were carried out using the B3LYP-D3 method with the 6–31 g (d, p) basis set. The electrostatic potential and molecular orbitals of the polymers were analyzed using Multiwfn software, and the resulting images were generated using the VMD program.

## 3. Results and Discussion

### 3.1. DFT Simulation and Analysis of PANI in Various Oxidation States

**Figure 3:**
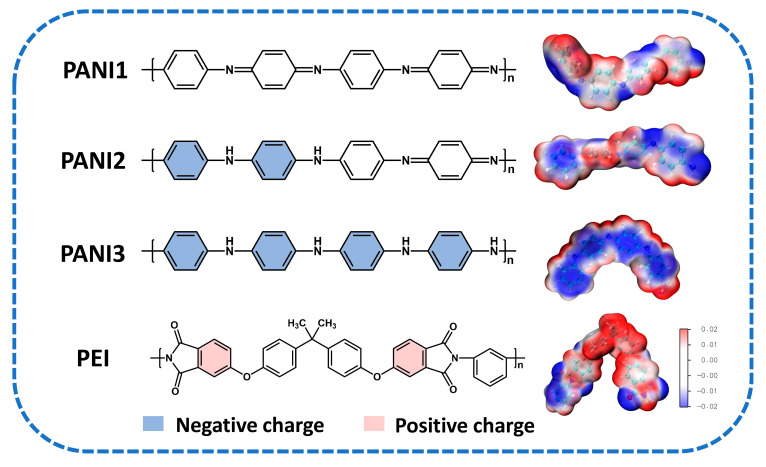
The molecular structure diagrams and 3D electrostatic potential simulation diagrams of PEI and PANI in different oxidation states, where red represents positive charges, and blue represents negative charges.

The molecular structure of PANI exhibits significant variations in the quinoid-to-benzoid ring ratio along its backbone at different oxidation states, which directly influences the surface electrostatic potential distribution under an applied electric field. To systematically investigate the electrostatic interactions between PANI at various oxidation states and the PEI polymer matrix, density functional theory (DFT) was employed to calculate and analyze the three-dimensional electrostatic potential distributions of fully oxidized polyaniline (PANI1), emeraldine base polyaniline (PANI2), fully reduced polyaniline (PANI3), and PEI, as shown in Figure 3. The structural units show that PANI1 contains the highest number of quinone rings in an alternating arrangement with benzene rings. PANI2 contains one quinoid ring and three benzoid rings, while PANI3 was modeled with an all-benzoid backbone and no quinoid rings to enhance comparative clarity. According to the calculated electrostatic potential maps, PANI exhibits fewer helical structures due to the absence of large side groups. The main chain of PANI3 appears distinctly blue, indicating that the benzene rings carry negative charges, as highlighted in blue in the structural diagram. Conversely, as the quinoid ring content increases, the blue tint diminishes, reflecting more irregular and dispersed negative charge distributions. PEI molecular chains are more coiled due to the strong intermolecular forces from the numerous nitrogen-containing five-membered heterocyclic and aromatic rings in the main chain. The phenyl groups linked to the imine moieties in PEI carry a positive charge, denoted in red in the structure, consistent with previously reported simulation results [39,40]. The theoretical results demonstrate that higher reduction degrees of PANI correlate with increased benzene ring content along the backbone, leading to stronger electrostatic interactions with PEI. In the fully reduced structure of PANI3, the high density and uniform distribution of benzoid rings yield a more negative electrostatic potential, indicating the most significant interaction with PEI.

**Figure 4:**
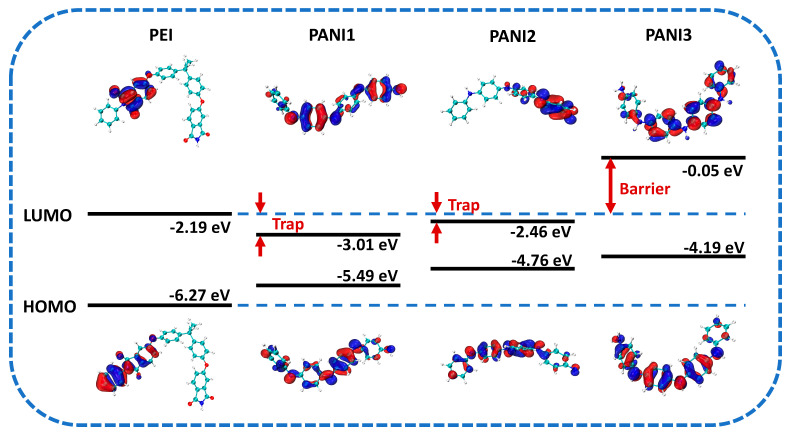
The energy level distribution and molecular orbital simulation diagrams of PEI and PANI in different oxidation states.

To investigate the band structures and interaction mechanisms between PANI in different oxidation states and the PEI matrix, the LUMO and HOMO energy levels and band gaps were simulated, and the electron transport characteristics and energy level alignment were analyzed. Figure 4 shows the theoretical simulation results of the molecular orbitals and band structures along with the energy level values. These values are in good agreement with the results measured by UV–vis absorption spectroscopy and ultraviolet photoelectron spectroscopy (UPS), confirming the reliability of the simulation. As the degree of reduction in PANI increases, both the LUMO and HOMO levels shift upward, indicating that PANI gradually exhibits electron-repulsion characteristics. Concurrently, the spatial redistribution of electron density reduces the molecule’s capacity to accept external electrons, thereby inhibiting electron conduction and trapping processes. When integrated into the PEI matrix, these redox-dependent energetic shifts critically modulate interfacial charge transfer dynamics. Specifically, PANI1 has a lower LUMO level and tends to capture electrons when incorporated into the PEI matrix, forming electron traps with a depth of 0.82 eV. Although the LUMO of PANI2 is higher than that of PANI1, it remains lower than that of PEI, so electrons in the PANI2/PEI system still tend to transfer from PEI to PANI2 and become trapped with a trap depth of 0.27 eV. Notably, the LUMO level of PANI3 is significantly higher than that of PEI, showing strong electron repulsion. Its introduction into the PEI system induces electron scattering effects [41] and forms an energy barrier as high as 2.14 eV at the PEI/PANI3 interface. Consequently, the mean free path of charge carriers is markedly shortened, impeding high-mobility transport and effectively enhancing dielectric breakdown strength while reducing conductive losses in the composite film.

### 3.2. Comparative Characterization Analysis of PANI and R-PANI

In the synthesis procedure designed in this section, PANI was prepared with gradient reaction times and then reduced under identical conditions. To elucidate the influence of reaction time on the molecular weight (M_w_) of R-PANI, gel permeation chromatography was performed on R-PANI-3, R-PANI-6, R-PANI-9, and R-PANI-12. The number-average molecular weight (M_n_), weight-average molecular weight (M_w_), and polydispersity index (PDI) of R-PANI are summarized in Table 2. The data reveal that both M_n_ and M_w_ initially increase and subsequently decrease as the reaction time increases. For instance, R-PANI-3, synthesized over 3 h, exhibits an M_n_ of merely 39,532, whereas R-PANI-9, obtained after 9 h of reaction, reaches a maximum M_n_ of 48,412. Further extension of the reaction time to 12 h (R-PANI-12) results in a reduction in M_n_ to 43,407. Coupled with the gradual increase in PDI over the reaction course, it is evident that excessively long reaction times lead to a non-uniform molecular weight distribution. Overall, R-PANI-9 demonstrates an optimal balance of high molecular weight and favorable molecular uniformity.

**Figure 5:**
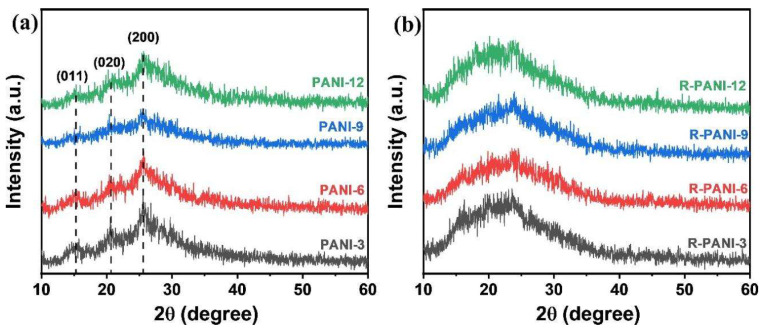
XRD patterns of PANI (**a**) and R-PANI (**b**) at different protonation times.

XRD and IR analyses were performed before and after reduction to compare the changes in crystallinity and molecular bonding. As shown in the XRD patterns of the four PANI samples in Figure 5a, all polymers synthesized with reaction times ranging from 3 to 12 h exhibit identical peak positions, corresponding to the (200), (020), and (011) crystalline phases at 25.6°, 20.7°, and 15.3°, respectively. This observation is consistent with previous reports [42,43], indicating that the polymerization time does not significantly affect the crystalline phase positions of PANI. Further examination reveals that PANI-9 displays the weakest diffraction intensity, which may be attributed to its higher molecular weight. The entanglement of high-molecular-weight chains and increased flexibility likely disrupt the crystalline order, resulting in a more pronounced amorphous character. In the R-PANI samples (Figure 5b), all diffraction peaks are broadened and shifted to lower angles, suggesting a decrease in crystallinity and an increase in interchain spacing. This phenomenon is presumably due to the partial removal of protons during reduction, which weakens the intermolecular interactions.

**Figure 6:**
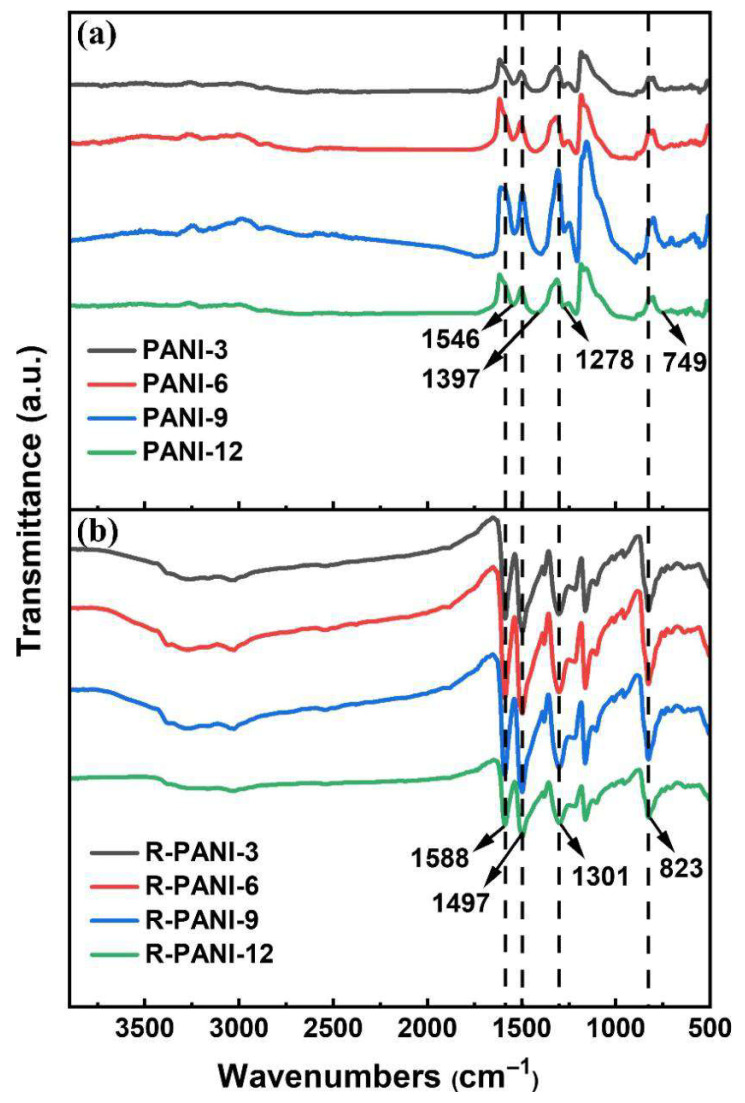
IR patterns of PANI (**a**) and R-PANI (**b**) at different protonation times.

Figure 6 presents the FTIR spectra of PANI synthesized at various polymerization durations, both before and after reduction. The polymerization time did not significantly alter the positions of the characteristic infrared absorption peaks of PANI, indicating that the basic structure of the polymer remained consistent under different polymerization times, but the positions of the infrared characteristic peaks before and after reduction underwent significant changes. Specifically, the characteristic peaks at 1546 cm^−1^ and 1397 cm^−1^ in PANI are attributed to the C=C stretching vibrations of the quinoid and benzenoid rings, respectively. The peak at 1278 cm^−1^ corresponds to the C–N stretching vibration in secondary aromatic amines, while the peak at 749 cm^−1^ represents the out-of-plane bending vibration of C–H in the benzene ring [31,34]. The presence of these peaks confirms the successful synthesis of PANI. After reduction, the characteristic peaks intensified and sharpened, exhibiting a shift toward higher wavenumbers: the C=C stretching vibrations of the quinoid and benzenoid rings appeared at 1588 cm^−1^ and 1497 cm^−1^, respectively; the C–N stretching vibration emerged at 1301 cm^−1^; and the C–H out-of-plane bending vibration shifted to 823 cm^−1^. This blue shift in the infrared absorption peaks can be ascribed to the de-doping process. De-doping causes the delocalized electrons generated during polymerization to relocalize, resulting in a more compact electron cloud distribution in the molecular structure. Consequently, vibrations of individual chemical bonds become more distinct and uniform, leading to a widening of the electronic transition band gap—consistent with the simulated band gap results shown in Figure 4. Moreover, the pronounced enhancement of the C–N stretching vibration in secondary aromatic amines and the C–H out-of-plane bending vibration in the benzene ring indicates an increase in reduced structural units along the polymer chains. This reflects the gradual conversion of quinoid rings into benzenoid structures, which disrupts the originally ordered crystalline phase and reduces the regularity of molecular chain packing. These observations further account for the decline in crystallinity of the R-PANI, as evidenced by the XRD patterns.

**Figure 7:**
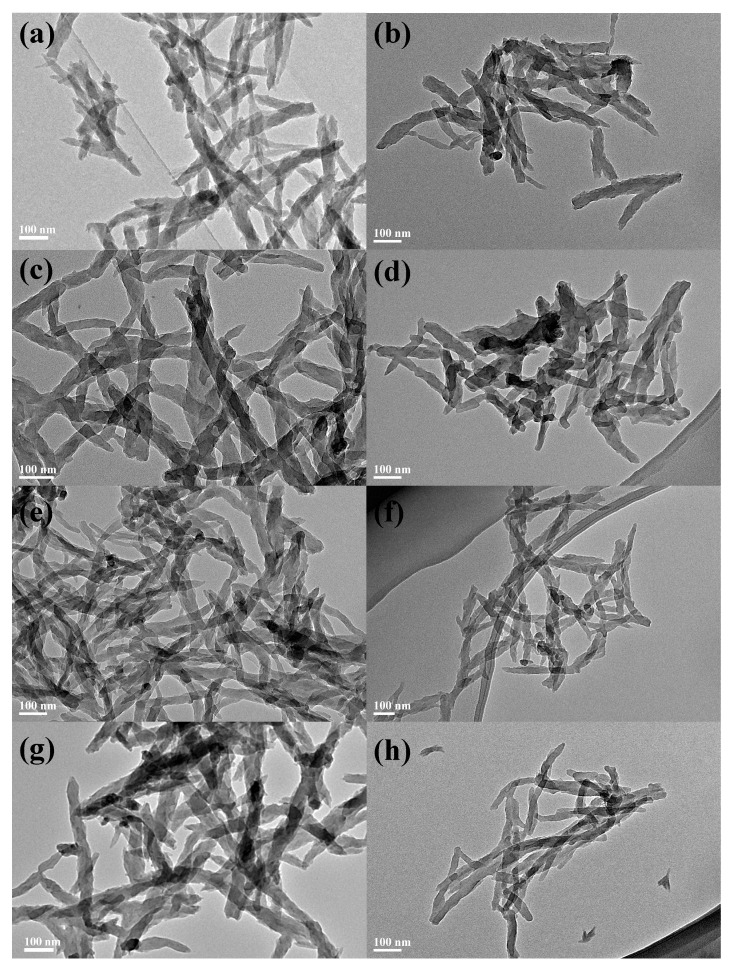
TEM images of ANI-3 (**a**), R-PANI-3 (**b**), PANI-6 (**c**), R-PANI-6 (**d**), PANI-9 (**e**), R-PANI-9 (**f**), PANI-12 (**g**), and R-PANI-12 (**h**).

**Figure 8:**
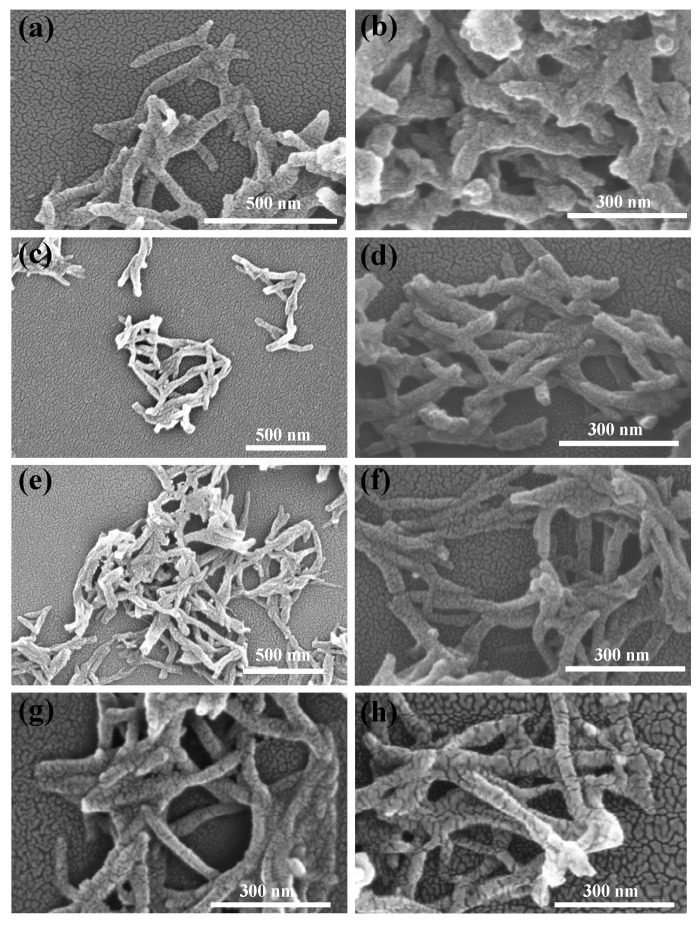
SEM images of ANI-3 (**a**), R-PANI-3 (**b**), PANI-6 (**c**), R-PANI-6 (**d**), PANI-9 (**e**), R-PANI-9 (**f**), PANI-12 (**g**), and R-PANI-12 (**h**).

For a more intuitive comparison of the morphological changes in PANI before and after reduction, as well as the influence of different polymerization durations on the PANI morphology, Figure 7 and Figure 8 present the transmission electron microscopy (TEM) and scanning electron microscopy (SEM) images of the four PANI and R-PANI samples. The images reveal that PANI exhibits an elongated morphology with lengths ranging from 200 to 1000 nm and widths of around 50 nm, indicating that the synthesized PANI possesses certain one-dimensional structural characteristics. R-PANI still maintains its elongated morphology with little discernible difference from its post-polymerization form, suggesting that the reduction process primarily alters the crystallinity and intermolecular interactions of the polymer without significantly affecting its growth morphology. Observations reveal increased flexibility in R-PANI samples with longer polymerization times (as shown in Figure 7f,h and Figure 8f,h), indicating that high molecular weight R-PANI possesses enhanced chain flexibility and looser arrangement. These changes are consistent with the reduced crystallinity observed in the XRD patterns after reduction.

### 3.3. Characterization of Dielectric Properties of R-PANI/PEI All-Organic Composite Films

**Figure 9:**
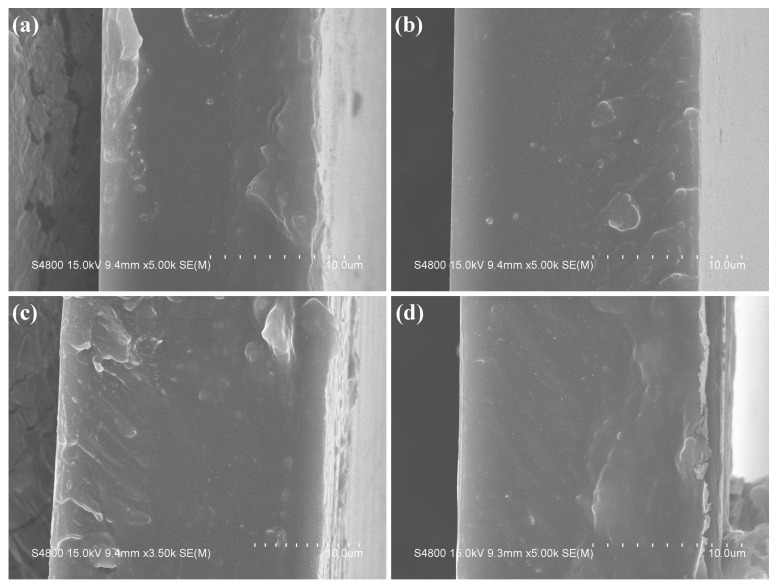
Cross-sectional SEM images of R-PANI-3/PEI (**a**), R-PANI-6/PEI (**b**), R-PANI-9/PEI (**c**), and R-PANI-12/PEI (**d**) at 1 wt% R-PANI contents.

The R-PANI samples synthesized at various polymerization durations were blended with PEI to form composite films. Prior to the dielectric performance investigation, a comparative analysis of their cross-sectional morphology was conducted. As illustrated in Figure 9, all films exhibited compact and dense structures without discernible voids, cracks, or delamination, indicating that effective compatibility between R-PANI and PEI was achieved through the blending method employed in this study. The molecular chains achieve mutual penetration due to their good dispersion in DMAc and the existence of electrostatic interactions and hydrogen bonding between the -NH groups of R-PANI and the imide or ether groups of PEI. The excellent compatibility of the two components provides a foundation for superior dielectric performance.

**Figure 10:**
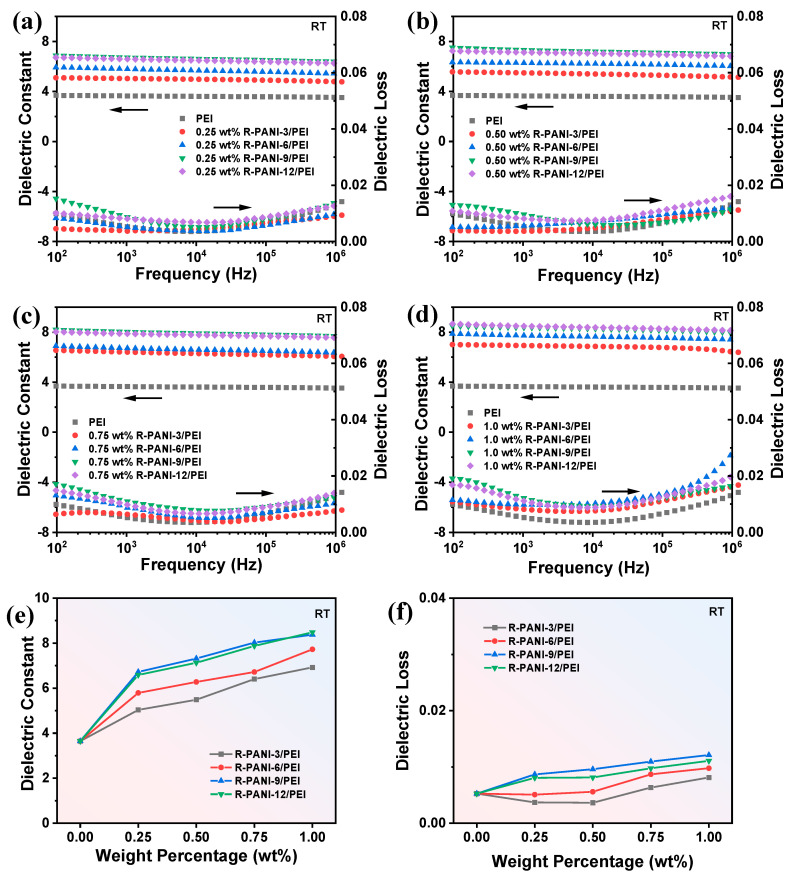
The dielectric constant and dielectric loss versus frequency at 0.25 wt% (**a**), 0.50 wt% (**b**), 0.75 wt% (**c**), and 1.0 wt% (**d**) for R-PANI/PEI under different protonation times; comparison of dielectric constant (**e**) and dielectric loss (**f**) for R-PANI/PEI at different weight percentages under 1 kHz.

To comprehensively investigate the effects of R-PANI with different molecular weights on the dielectric constant and dielectric loss of PEI composite films, four types of R-PANI were blended with PEI at various mass fractions, and the dielectric properties were measured as shown in Figure 10. Based on the variations in dielectric constant and dielectric loss with frequency and R-PANI mass fraction, the following conclusions can be drawn. Firstly, at the same mass fraction, R-PANI-3/PEI consistently exhibits the lowest dielectric loss, whereas R-PANI-9/PEI demonstrates the highest dielectric loss. Considering the difference in M_w_ between the two, it can be inferred that shorter-chain molecules facilitate more homogeneous dispersion within the PEI matrix, leading to faster polarization response under an applied electric field, thereby minimizing dielectric loss, particularly at high frequencies. In contrast, although higher molecular weight segments possess greater chain flexibility, longer molecular chains form more polarization centers at the interfaces, which may result in increased dielectric loss. Given that interfacial polarization typically responds at low frequencies, the higher dielectric loss observed at lower frequencies further corroborates this mechanism. Conversely, at the same mass fraction, R-PANI-9/PEI consistently achieves the highest dielectric constant, while R-PANI-3/PEI exhibits the lowest dielectric constant. This is because longer and more flexible molecular chains enhance the polarization response of the segments and create more polarization regions under an electric field. In comparison, short-chain molecules exhibit weaker polarization response and less pronounced interfacial polarization effects than high molecular weight R-PANI, leading to a lower dielectric constant. The most significant disparity in the dielectric constant of R-PANI-9/PEI between low and high frequencies further validates the above interpretation.

Compared with pure PEI, the addition of R-PANI significantly increases the dielectric constant regardless of the molecular weight. Moreover, the dielectric constant increases progressively with higher R-PANI loading, demonstrating the efficacy of R-PANI in improving the polarization capability of the composite films. Meanwhile, the dielectric loss of the composites does not exhibit a notable increase relative to pure PEI. In particular, the R-PANI-3/PEI composite even exhibits reduced dielectric loss at low filler content. This observation indicates that the prepared R-PANI becomes increasingly insulating following the de-doping process, and the electron transport barriers established by R-PANI in PEI suppress conduction loss. Furthermore, an analysis of the variations in dielectric constant and dielectric loss with R-PANI mass fraction, as illustrated in Figure 10e,f, suggests that excessive R-PANI loading should be avoided. High R-PANI content may lead to molecular chain overlapping, particularly at high molecular weights, where entangled chains impede dipole orientation under an electric field, consequently resulting in increased dielectric loss [29].

**Figure 11:**
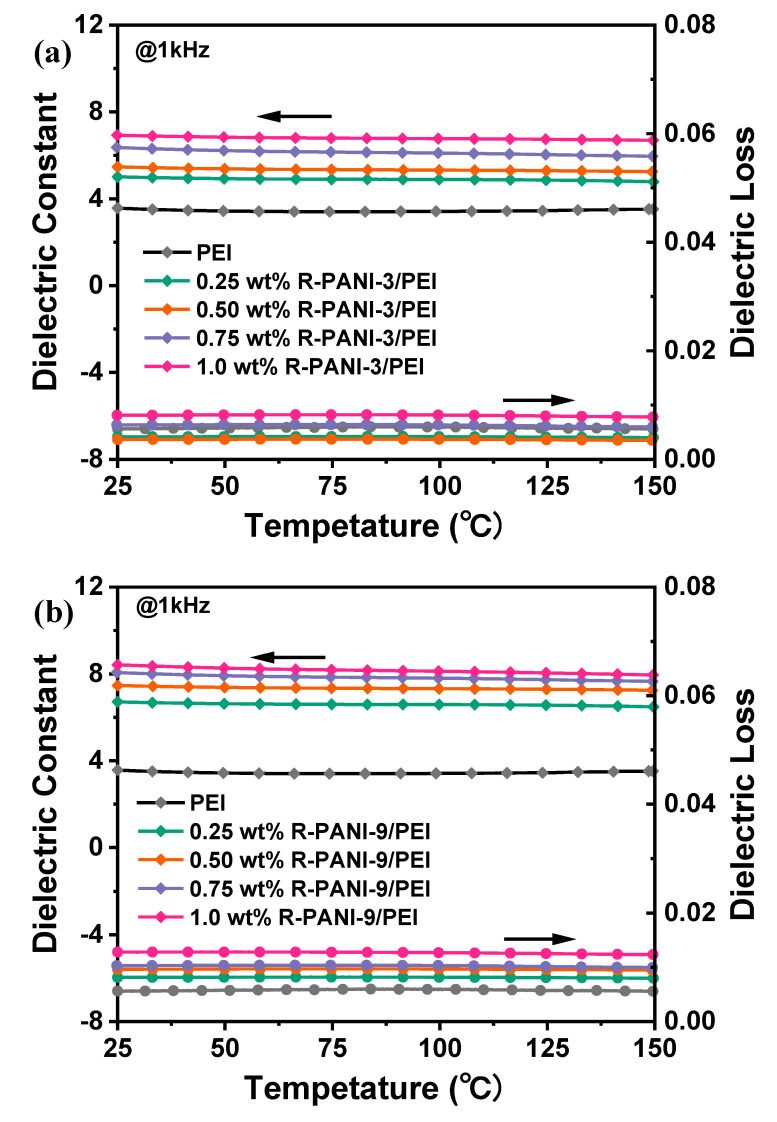
Temperature-dependent curves of dielectric constant and dielectric loss for R-PANI-3/PEI (**a**) and R-PANI-9/PEI (**b**) with different R-PANI contents.

Furthermore, the temperature dependence of the dielectric constant and dielectric loss for R-PANI-3/PEI and R-PANI-9/PEI composite films was measured from 25 to 150 °C at a frequency of 1 kHz, as shown in Figure 11. Evidently, the high-temperature dielectric stability of R-PANI/PEI composites exhibits minimal variation in both dielectric constant and loss at elevated temperatures. This is primarily attributed to the intrinsic high-temperature dielectric stability conferred by the substantial amount of PEI. Moreover, despite active carrier and molecular chain movements at high temperatures, the polarization and carrier migration behaviors show no significant changes due to the existence of carrier migration energy barriers and electrostatic interactions between molecular chains.

**Figure 12:**
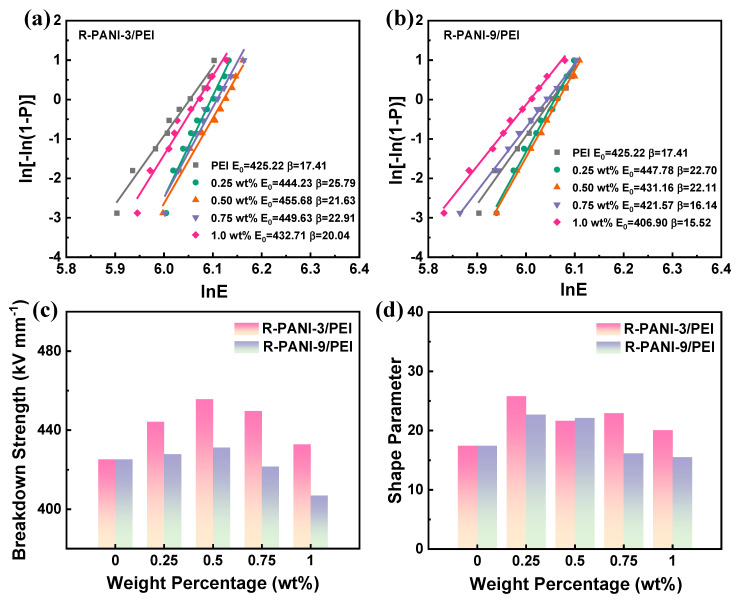
Weibull distribution of the measured breakdown strength for R-PANI-3/PEI (**a**) and R-PANI-9/PEI (**b**) at different loadings; comparisons of the characteristic breakdown strength (**c**) and shape parameter (**d**) for R-PANI-3/PEI and R-PANI-9/PEI at various loadings.

To further compare the dielectric performance of R-PANI-3/PEI and R-PANI-9/PEI composite films with different molecular weights, the breakdown strength of the two composite systems was measured at varying mass percentages, as shown in Figure 12a,b. The obtained breakdown strength data followed the Weibull distribution as described in Equation (1), and the characteristic breakdown strength E_0_ and shape parameter β derived from the fitting procedure are summarized in Figure 12c and Figure 12d, respectively. Comparative analysis reveals that the incorporation of R-PANI-3 significantly enhances the breakdown strength of the PEI matrix, primarily due to the electronic energy barrier introduced by R-PANI-3 and the electrostatic interactions with the phenyl rings in the PEI backbone. At the same mass fraction, the breakdown strength of R-PANI-3/PEI consistently exceeds that of R-PANI-9/PEI, which is closely associated with its lower dielectric loss. Materials with lower dielectric loss generate less thermal accumulation under an electric field, thereby reducing the risk of thermal degradation-induced breakdown. The shape parameter of R-PANI-3/PEI also remains at a high level because short-chain R-PANI-3 distributes more uniformly in the PEI matrix, leading to consistent breakdown strength and enhancing overall electrical performance and reliability.

(1) $$P(E)\;=\;1-exp[-(\frac{E}{E_{0}}{)}^{\beta }]$$

Interestingly, while the dielectric loss of R-PANI-9/PEI is higher than that of the PEI matrix, the breakdown strength does not significantly decrease at low filler loadings. However, when the filler content exceeds 0.5 wt%, both the breakdown strength and shape parameter decline concurrently. This indicates that long-chain R-PANI tends to undergo chain entanglement and form microscopic defects at higher loadings. These defects can act as localized electric field concentration sites, leading to a reduction in breakdown strength, whereas at low loadings, the electronic transport barriers introduced by R-PANI contribute to the maintenance of high breakdown performance. First, our findings reveal that the increase in characteristic breakdown strength $E_{0}$ is mainly due to the electronic barrier effect introduced by R-PANI, which effectively inhibits carrier migration and delays the initiation of electrical breakdown. Second, it is noticeable that the change in shape parameter β directly reflects the homogeneity and defect state inside the material. The low molecular weight R-PANI3 significantly reduces micro defects due to its short chain, uniform dispersion, and strong electrostatic/hydrogen bond interaction with the PEI matrix, so the β value is higher, indicating that the composites have a narrower failure distribution and higher reliability, and vice versa. All these analyses confirm a relationship between the Weibull parameter and microstructure.

### 3.4. Characterization of Energy Storage Performance for All-Organic R-PANI/PEI Composite Films

**Figure 13:**
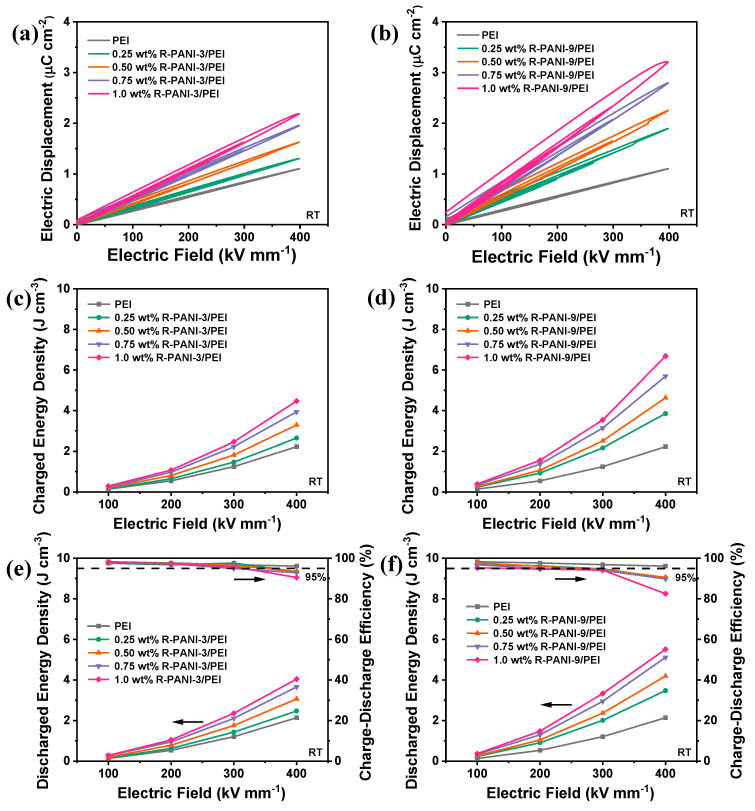
D–E loops of R-PANI-3/PEI (**a**) and R-PANI-9/PEI (**b**) at different mass contents; charged energy density of R-PANI-3/PEI (**c**) and R-PANI-9/PEI (**d**) under different electric fields; discharge energy density and charge–discharge efficiency of R-PANI-3/PEI (**e**) and R-PANI-9/PEI (**f**) under different electric fields.

To investigate the specific effects of R-PANI-3 and R-PANI-9 on the energy storage performance of PEI composite films, the D–E charge–discharge loops of R-PANI-3/PEI and R-PANI-9/PEI composite films with graded mass fractions were measured, as shown in Figure 13a,b. Comparative analysis reveals that at the same doping level, the discharge curves of R-PANI-9/PEI are broader than those of R-PANI-3/PEI, indicating higher energy loss during the charging–discharging process in the former. This is likely due to the significant dipolar hysteresis of the long chain segments under an applied electric field, which increases energy loss and leads to decreased efficiency. In contrast, shorter molecular chains facilitate more flexible dipole reorientation under electric fields, thereby reducing energy loss and charge hysteresis. Moreover, the shorter chains promote a more homogeneous distribution within the matrix, which minimizes conductive pathways and local defects. This observation is consistent with the higher breakdown strength observed for R-PANI-3/PEI in Figure 12. However, the electric displacement intensity of R-PANI-9/PEI is generally higher than that of R-PANI-3/PEI at the same loading. This is due to the greater flexibility and polarization capability of the longer polymer chains, which can form more polarization domains under an electric field, thereby leading to an overall enhancement in electric displacement intensity. This result also aligns with the higher dielectric constant observed for the former in Figure 10.

Furthermore, the charging energy density (Figure 13c,d), discharging energy density, and charge–discharge efficiency (Figure 13e,f) of the two composite films were calculated at electric fields ranging from 100 to 400 kV·mm^−1^. It is evident that the electric displacement intensity of both systems increases with higher doping levels, resulting in a continuous rise in charging energy density, significantly surpassing that of pure PEI. At low electric fields, R-PANI-3/PEI with low loading even achieves a higher efficiency than pure PEI, confirming the lower dielectric loss and demonstrating that the electronic potential barriers introduced by R-PANI3 effectively reduce energy dissipation from conduction loss. Under a 400 kV mm^−1^ electric field, the discharge energy density of 1.0 wt% R-PANI-3/PEI reached 4.04 J cm^−3^, with a charge–discharge efficiency as high as 90.5%. Meanwhile, the discharge energy density of 1.0 wt% R-PANI-9/PEI reached an even higher 5.51 J cm^−3^, with a charge–discharge efficiency of 82.5%, both of which were higher than the 2.14 J cm^−3^ discharge energy density of pure PEI. At a high charge–discharge efficiency of over 95%, the maximum discharge energy density that R-PANI-3/PEI could achieve was 2.36 J cm^−3^, with a doping amount of 1.0 wt% and an electric field strength of 300 kV mm^−1^. For R-PANI-9/PEI, the maximum discharge energy density of 1.29 J cm^−3^ was reached at 200 kV mm^−1^, with a doping amount of 0.5 wt%.

**Figure 14:**
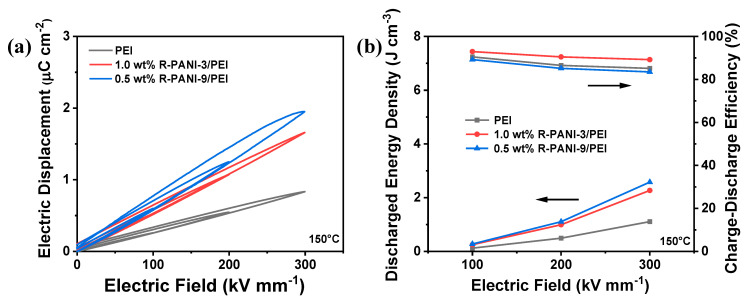
D–E loops (**a**) and discharge energy density and charge–discharge efficiency under different electric fields (**b**) of PEI; 1.0 wt% R-PANI-3/PEI, and 0.5 wt% R-PANI-9/PEI at 150 °C.

The energy storage performance of 1.0 wt% R-PANI-3/PEI, 0.5 wt% R-PANI-9/PEI, and pure PEI was further compared at 150 °C (Figure 14). The results indicate that all three systems experienced increased energy loss and reduced charge–discharge efficiency under elevated temperature, which is primarily attributable to enhanced charge carrier mobility under such conditions. Notably, R-PANI-3/PEI composite maintained relatively high charge–discharge efficiency, suggesting that the energy barrier for electron migration remains effective and plays an even more significant role at high temperatures. Under an electric field of 300 kV mm^−1^, the 1.0 wt% R-PANI-3/PEI composite achieved a discharge energy density of 2.27 J cm^−3^ while retaining a high charge–discharge efficiency of 89.2%. In contrast, under the same conditions, pure PEI exhibited an efficiency of only 85.1% and a discharge energy density of 1.11 J cm^−3^.

### 3.5. Carrier Transport Mechanisms Within R-PANI/PEI All-Organic Composite Thin Films

**Figure 15:**
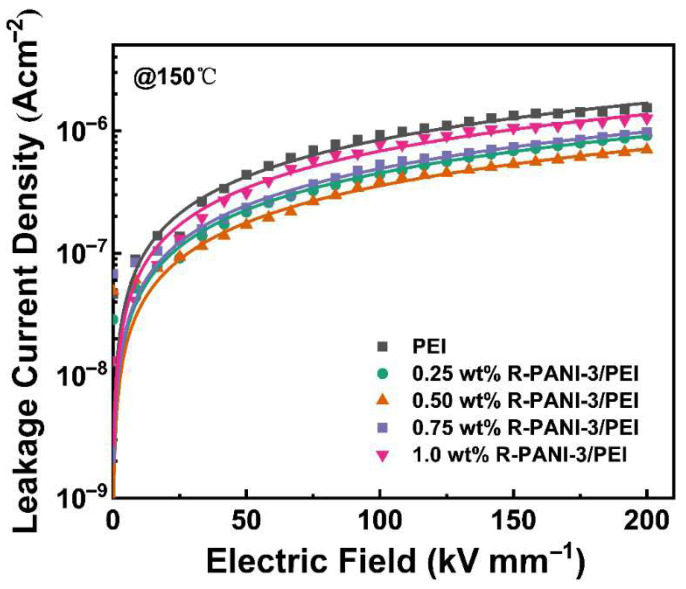
Leakage current density curves of R-PANI-3/PEI films under different mass contents at 150 °C.

To elucidate the specific impact of R-PANI3 incorporation into PEI on charge carrier transport, the leakage current density of R-PANI-3/PEI thin films with gradient concentrations ranging from 0 to 1 wt% was compared under conditions of 150 °C and an electric field of 0–200 kV mm^−1^. As illustrated in Figure 15, the data were well-fitted by the hopping conduction equation described earlier. The leakage current density of all R-PANI-3/PEI films was lower than that of pure PEI, indicating that the inclusion of R-PANI-3 in PEI can impede carrier migration at elevated temperatures, leading to reduced energy loss. This observation aligns with the higher charge–discharge efficiency exhibited by the 1 wt% R-PANI3/PEI composite compared to pure PEI, as shown in Figure 14. Moreover, 0.5 wt% R-PANI-3/PEI shows the lowest leakage current density, while further increasing the loading leads to higher leakage current, suggesting that high doping levels weaken the inhibition effect. This phenomenon is attributed to the potential aggregation of R-PANI3 molecular chains at higher doping levels, which may form conductive pathways or microscopic defects, causing local electric field concentration and increasing both leakage current and dielectric loss. Overall, DFT predicts that there is an electronic barrier as high as 2.14 eV between R-PANI3 and PEI (Figure 4), which directly explains the phenomenon that the leakage current density is significantly reduced (Figure 15) and the breakdown strength is increased (Figure 12) observed in the experiment. According to GPC data (Table 2), R-PANI-3 is characterized by a low molecular weight sample (M_n_ = 39,532) while R-PANI-9 exhibits a relatively high molecular weight sample (M_n_ = 48,412). The low molecular weight of R-PANI-3 mainly enables a strong electronic barrier, depending on good dispersion and few defects, significantly improving its breakdown strength (Figure 12) and suppressing the leakage current (Figure 15). Comparatively, the high molecular weight of R-PANI-9 mainly leads to high dielectric polarization due to its stronger polarization response in the long chain segment, resulting in discharge efficiency, with 5.51 J/cm^3^ at 400 kV mm^−1^ (Figure 13), and slightly higher dielectric loss.

**Figure 16:**
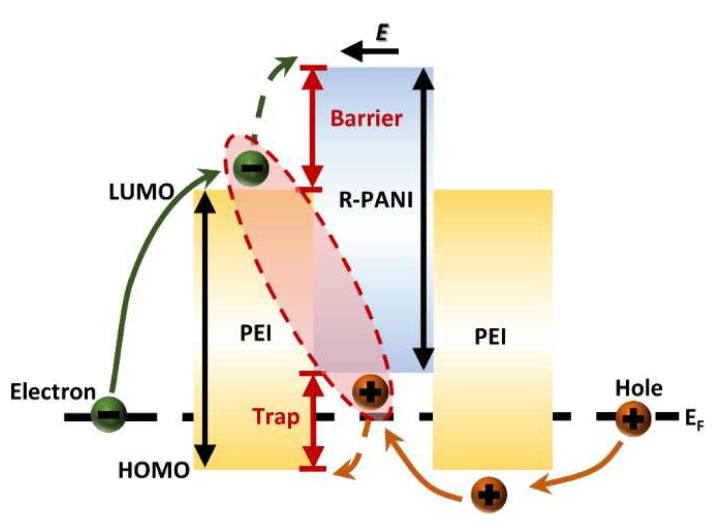
The schematic diagram of the energy band structure in the interfacial region of R-PANI-3/PEI composite film.

**Figure 17:**
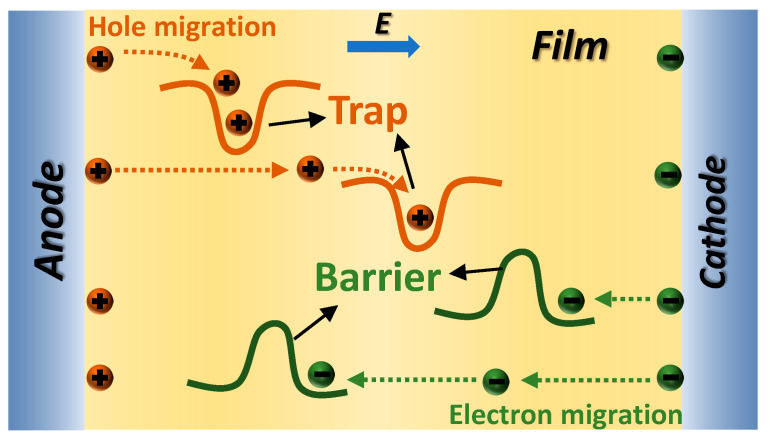
The schematic diagram of suppressed carrier migration within the R-PANI-3/PEI composite film.

The blocking mechanism of R-PANI3 on charge carriers within PEI can be elucidated by analyzing the energy band structure at the R-PANI3/PEI composite film interface in Figure 16. As previously indicated by simulations of the molecular orbitals of R-PANI and PEI, with increasing reduction degree of PANI, both the LUMO and HOMO energy levels of R-PANI gradually rise, thereby enhancing its electron-repelling properties. Under an applied external electric field, electrons and holes migrate in opposite directions. After electrons transfer to the R-PANI3/PEI interface, the energy level difference between the LUMO of R-PANI3 and that of PEI creates an electronic potential barrier, preventing further electron migration into R-PANI3. This impedes the formation of conductive pathways by electrons (Figure 17), while the higher HOMO energy level of R-PANI3 allows holes to easily transfer from PEI to R-PANI-3. These holes are captured by the hole traps created by the HOMO level difference and struggle to return to the PEI matrix (Figure 17). Furthermore, the electrons confined in the LUMO of PEI and the holes trapped in the HOMO of R-PANI3 can form electron–hole pairs, further restricting the migration of charge carriers. These combined effects contribute to the reduction in leakage current density achieved by appropriate doping with R-PANI3.

In summary, our research has achieved progress in the field of PANI-based energy storage materials, and we effectively avoid the traditional design paradigm that only contains inorganic fillers. In this study, we innovatively create an all-organic R-PANI/PEI composite system, which fundamentally solves the tough issues such as inorganic/organic interface thermal mismatch and filler agglomeration. By accurately adjusting the molecular weight and chain structure of reduced polyaniline, we construct an electron barrier hole trap strategy by defining the energy level difference to inhibit carrier migration. Comparing to other recent studies, we have overall advantages when considering from the aspect of the dielectric performance and temperature stability [27,28,29,30,31,32,33]. Our work provides a new idea based on molecular design for long-life high-temperature energy storage devices.

## 4. Conclusions

This study investigates the distinct dielectric responses of quinoid and benzenoid rings in the PANI backbone, aiming to simultaneously enhance the dielectric constant and reduce the dielectric loss of the PEI matrix through all-organic composite films of R-PANI with different molecular weights. By exploring the influence of molecular weight on dielectric properties, the discharge energy density of PEI-based polymers was significantly improved while maintaining high charge–discharge efficiency. Through DFT calculations and experimental validation, it is confirmed that reduced units facilitate stronger electrostatic interactions between PANI and PEI, while introducing higher electronic energy barriers and deeper hole traps to suppress charge carrier migration. Consequently, all PANI samples were chemically reduced with N_2_H_4_·H_2_O prior to incorporation into PEI. The experimental results reveal that R-PANI samples of varying molecular weights exhibit distinct advantages within the PEI matrix. High molecular weight R-PANI, due to its superior chain flexibility, promotes polarization and contributes to a larger interfacial polarization region, thereby increasing the dielectric constant. However, its tendency toward chain entanglement increases dielectric loss. In contrast, low molecular weight R-PANI displays better dispersibility, more effectively forming electronic barriers that reduce leakage current and lower dielectric loss. With charge–discharge efficiency maintained above 95%, R-PANI3/PEI achieved a discharge energy density of 2.36 J cm^−3^ at room temperature, nearly double that of pristine PEI (1.2 J cm^−3^). At 150 °C, the 1.0 wt% R-PANI3/PEI composite film retained a discharge energy density of 2.27 J cm^−3^ with a charge–discharge efficiency of 89.2%, outperforming pure PEI (1.1 J cm^−3^, 85.1%). This demonstrates that R-PANI significantly enhances dielectric performance and maintains excellent high-temperature energy storage due to its high electronic barriers and hole traps. This study offers a new direction for the design of all-organic dielectric composite films and highlights the potential of R-PANI for applications in high-performance capacitors and advanced electrical energy storage systems.

## Figures and Tables

**Table 1:** Materials and reagents.

Name	Specification	Manufacturer/Supplier
Aniline (AN)	≥99.9%	Shanghai Macklin Biochemical Co., Ltd.
Ammonium persulfate (APS)	≥99.99%	Shanghai Aladdin Biochemical Technology Co., Ltd.
Hydrochloric acid (HCl)	36.0–38.0%	Sinopharm Chemical Reagent Co., Ltd.
Hydrazine hydrate (N_2_H_4_·H_2_O)	80%	Shanghai Lingfeng Chemical Reagent Co., Ltd.
N,N-dimethylacetamide (DMAc)	99.5%	Shanghai Macklin Biochemical Co., Ltd.
Polyetherimide (PEI)	ULTEM 1000	BASF (China) Co., Ltd.

**Table 2:** Molecular weight and molecular weight distribution of R-PANI at different protonation times.

Samples	M_n_	M_w_	PDI
R-PANI-3	39,532	39,532	1.92
R-PANI-6	45,704	89,064	1.95
R-PANI-9	48,412	94,644	1.95
R-PANI-12	43,407	89,073	2.05

## Data Availability

The raw data supporting the conclusions of this article will be made available by the authors on request.
